# Design and Validation of an Observational Instrument for the Technical-Tactical Actions in Singles Tennis

**DOI:** 10.3389/fpsyg.2018.02418

**Published:** 2018-12-03

**Authors:** Gema Torres-Luque, Ángel Iván Fernández-García, David Cabello-Manrique, José María Giménez-Egido, Enrique Ortega-Toro

**Affiliations:** ^1^Faculty of Education, University of Jaen, Jaen, Spain; ^2^Department of Physiatry and Nursing, Faculty of Sports and Health Sciences, University of Zaragoza, Zaragoza, Spain; ^3^Department of Physical Education and Sport, Faculty of Sport Science, University of Granada, Granada, Spain; ^4^Department of Physical Activity and Sport, Faculty of Sport Science, University of Murcia, Regional Campus of International Excellence “Campus Mare Nostrum”, Murcia, Spain

**Keywords:** performance, evaluation, tennis, match analysis, observational methodology

## Abstract

The competitive performance in tennis practice is determined by the effectiveness of technical tactical action. The main objective of the present study was to design and validate an observational instrument with the aim of analysing the technical-tactical in singles tennis. The instrument uses the stroke as a unit of measure, so that each time a player hits a ball, a total of 23 variables are analyzed. The variables collect information about: (a) matching context; (b) result; and (c) technical-tactical information of the stroke (five variables: sequences of the stroke of the point, kind of technical and tactical stroke, bounce area, hitting, and effectiveness area). The design and validation of the instrument consisted on five different stages: (a) review of the scientific literature and variables definition by experts, (b) pilot observation study, (c) qualitative and quantitative assessment of the instrument by experts, (d) review and confirmation of the instrument by experts (content validity), and (e) observation training and reliability evaluation. From 23 expert judges, divided into three panels, and four observers the instrument went from being composed of 38 variables (eight contextual, seven related to the result and 23 related to the game) to 23 (eight contextual variables, 10 of result and five of game), with minimum Aikens's V values of 0.94 and reliability of 0.81. The results show that the designed instrument allows obtaining valid and objective information about the technical-tactical actions of the players and their performance in singles tennis

## Introduction

One of the most determining factors in sports performance is the tactical technical analysis of the competition (Cui et al., 2018; Zhang et al., 2018). This type of analysis allows coaches to obtain information about the performance indicators, apart from knowing the weaknesses and strengths from their own and the adversaries, offering the possibility of improving training processes and therefore, increasing the possibilities of performance and success possibilities from the players (O'Donoghue and Ingram, 2001; Sainz De Baranda et al., 2008). That is why many coaches and researchers use the technological advances and the different methods of sport science for this purpose (Figueira et al., 2018).

Despite the multiple and quick advances in technology (Mateus et al., 2017), observation processes are still a very important instrument when it comes to gathering information in the sports field. Proof of this is that one of the methods most used by researchers in the last decade in the field of match and notational analysis is the observational methodology (Anguera et al., 2011; Maneiro et al., 2018). Thanks to it, the multiple variables that concur and interact in motor competition can be registered, as well as all those related to the context in which they are developed, such as the type of activity (competition or training), the level of expertise (professionals or amateurs) or the different categories that exist in each sport (Anguera and Hernández-Mendo, 2015).

One of the essential aspects in any type of scientific methodology is the recording of data through an appropriate instrument that guarantees its reliability and validity (Chacón-Moscoso et al., 2018). According to Hughes et al. (2002) 70% of the papers presented in conferences in which the observational methodology is used in sports, have deficiencies in data collection and statistical treatment. This is why, in the sports field, different studies can be found which objective has been to validate a specific observational instrument from of a sport modality, such as those carried out in handball (Morillo et al., 2017), rugby (Jones et al., 2008; Villarejo et al., 2014), beach volleyball (Palao et al., 2015), basketball (Moreno and Gómez-Ruano, 2017), soccer (Larkin et al., 2016; Maneiro et al., 2018) etc. All of them describe the process in two large phases: (a) design of the instrument with the system of categories and behaviors to observe; (b) to establish and calculate the reliability and validity of the instrument.

In the case of tennis, many investigations have used observational methodology in the match and notational analysis to obtain information about aspects such as performance indicators (Djurovic et al., 2009; Katić et al., 2011; Cui et al., 2018), service effectiveness and returns (Gillet et al., 2009; Hizan et al., 2011; Martin et al., 2016), tactical aspects (Over and O'Donoghue, 2010; Cross and Pollard, 2011; Reid et al., 2016), or the displacements and the position of players on the tennis court (Martínez-Gallego et al., 2013; Pereira et al., 2017). Sometimes these data are downloaded directly from the official websites of the tournaments and in others, the researchers are the ones who perform the data collection through observation. However, in none of the cases the process of design and validation of the instrument that they have used to collect the information are explained.

Therefore, the purpose of this article was to design and validate an observational instrument that allows coaches and researchers to analyse in a reliable, objective, accurate, and valid way the technical tactical actions in singles tennis.

## Methods

The design and validation of the observational instrument was carried out in five phases. In the first two, the design of the observational instrument was carried out, which consisted of a system of categories (Anguera, 2003; Anguera and Hernández-Mendo, 2015). In the third and fourth, the content validity was established and calculated, while in the fifth phase the reliability of the instrument was tested (Kinrade et al., 2010).

The objective of the first phase was to prepare a provisional list of the behaviors to be studied once the review of the scientific literature has been carried out (in the Sport Discus, Web of Science, Google Scholar, Sponet, Scielo, and Dialnet databases) with the keyword single tennis and excluding (not) the word table (Table 1). The result was an initial list of variables that included the definition of each of the variables and the category to which they should belong. The unit of analysis was the tennis stroke and three categories were established to group all the variables: (a) contextual variables, (b) result variables, (c) game variables. In the first group were included all those that defined the environment of a match. In the second, the variables that gave information about the score of the match were included, while in the third one there were introduced those related to the execution, result, and effectiveness of the technical-tactical actions. The provisional list of variables and categories was presented and analyzed by three experts who had at least the following characteristics: (a) have a minimum qualification of Sports Technician of the highest national category; (b) have a minimum experience of 10 years in the teaching of tennis; and (c) be graduated in Physical Activity and Sports Sciences. So that there was no modification of variables and categories, all of them had to, by consensus, give their approval to all of them.

**Table 1:** Questionnaire example sent to the experts.

**Stroke effectiveness**
•Variable: Effectiveness of the stroke performed by the player.
•Categories:
1. Winner. Stroke made by the player with the one that gets the point directly, without his/her opponent touched the ball.
2. Transition stroke. Stroke made by player after that, the opponent hit the ball and bounce inside the court of the first one.
3. Previous stroke of an opponent error. Stroke made by player after that, the opponent hitting the ball and committed an error losing the point.
4. Error. The player hit the ball sending out of the regulatory area of the court or to the net losing the point.
**a) Inclusion:** Do you consider it necessary to include this variable in the observation sheet? YES/NO
**b) Adequacy:** Do you think that the definition of the variable and its categories is adequate?
• Very inadequate 1-2-3-4-5-6-7-8-9-10 very suitable
• In the case that it would be necessary to add or delete a category, indicate which one and why.
**c) Writing:** Do you consider adequate the wording of the definition of the variable and the definition of each of the categories?
• Very poorly written 1-2-3-4-5-6-7-8-9-10 very well written
• Propose a definition if it is not clear:
**d) Observations:**

*Variable stroke effectiveness*.

In the second phase, a pilot observation was carried out to test the previously formed instrument with the objective of making modifications if deemed necessary. To do so, a single observer independent in relation to the investigation carried out, analyzed all the seven-set shots from three matches of the 2014 Tennis Masters Cup. The observer had a degree in sports science, had more than 10 years' experience as a tennis coach and had the highest degree sports as a national tennis coach. The analysis and its corresponding report was reviewed by the researchers and experts previously consulted, with the aim of generating a second list of variables and categories with the modifications carried out with respect to the previous phase. This way observation is used to add sports behaviors that previously were not defined at a theoretical level, but which appeared during the game. Similarly in this phase the frequencies that occur in some categories are observed, with a view to use this data to delimit and define the size of the categories (for example after the study of the service and from the number of actions, it was divided the serve bounce areas in those noted in the instrument and no more or less areas). In this phase, an observation manual was created, in which all the variables and their categories were named and defined. On the other hand, the recording (annotation) of the information was done manually in the Excel statistical program.

In the third phase, a quantitative and qualitative evaluation of the instrument was carried out by another 10 new experts who should have the following characteristics (federated tennis coach, graduated in Physical Activity and Sports Sciences, and at least 10 years of experience as a coach). In this new evaluation the expert judges completed a questionnaire in which they were asked about each of the variables under study, including the following aspects: (a) convenience of including the behavior or variable in the observational instrument (Inclusion); (b) degree of adequacy in the definition of the variable and the categories that compose it (Adequacy); (c) level of writing of the definitions of the variable and of the categories that were part of the instrument (Writing); and (d) observations. The quantitative part of the evaluation consisted in scoring from 1 to 10 the adequacy and definition part and the qualitative part in answering with “Yes” or “No” the inclusion section. The part of observations would be completed in the case that it is considered necessary to make a contribution. The data were recorded and a descriptive analysis was made (mean, median and mode of each continuous variable and absolute and relative frequency of the categorical variables).

Later on, the content validity was calculated through of Aikens's V coefficient (Aiken, 1980; Penfield and Giacobbi, 2004). The Visual Basic 6.0 software application of Merino and Livia (2009) was used. To define the criteria for elimination or modification, the of Aikens's V coefficient (Aiken, 1985) was applied. A critical level of Aikens's V was established to reject the null hypothesis, obtaining a value of 0.70 (*p* = 0.05) and 0.81 (*p* = 0.01). From these values, it was decided to eliminate the items with values lower than 0.70 and to modify the items with values between 0.70 and 0.81. The items with higher values than 0.81 were maintained. Likewise, it was considered as a minimum value for inclusion that at least 80% of the expert judges answered yes, in the inclusion question.

After the modifications made in phase three, in the fourth phase a new qualitative and quantitative analysis of the instrument was carried out to another 10 experts, who fulfilled identical characteristics to the previous ones, not repeating in any case. A descriptive analysis of all the variables (mean, median, mode, and frequencies) was carried out and the validity of the content was calculated by means of an of Aikens's V coefficient (Penfield and Giacobbi, 2004). Finally, a new and definitive list of variables and categories was created that led to the writing of the observation instrument (“Observation instrument for singles tennis” see Supplementary Data Sheet 1) that included the variables under study and their definition, together with all the categories that were grouped in each of them, their definitions and coding.

In the fifth phase the reliability of the instrument was tested, as it was done in other studies (Villarejo et al., 2014; Gamonales et al., 2018). Following the criteria of Anguera (2003) and Losada and Manolov (2015), three observers received a training led by the principal investigator consisting of three sessions of 2 h each with a break of 10 min once they reached the 55 from the observation. To do so, the observation manual designed in the fourth phase was used. To assess the reliability, three experts (graduates in Physical Activity and Sports Sciences and federated tennis coaches) evaluated each of them, twice, separated by a week, two sets of two men's tennis matches. For the inter-observer and intra-observer calculation Cohen's Kappa coefficient was used, recording the lowest value.

Last but not least, it's necessary to develop the protocol for the correct use of the instrument. In the first place, the view of the observer must be made behind from any baseline of the tennis court in an elevated position, which allows watching clearly all the lines (the minimum height would be above the head of the players). If the video recording is made using a video camera, the previous guidelines will be followed. The collection of observational data in relation to the variables of the bounce area and hitting area will be done according to the zones indicated in the fields of Supplementary Data Sheet 1.

The data will be recorded by means of an Excel spreadsheet previously designed, where each row is a stroke to observe the previous action of the adversary, the action of the player and the consequence of his stroke on the adversary, which will perform another action in response (see Figure 1). The objective of this record mode is to know the sequence of strokes in the interaction between player and opponent.

In order to optimize the recording time of all the variables and their categories, the order will be explained below at a temporal level: (a) variables to record each stroke [RESULTS (Game scoreboard and Winner or loser of the analyzed point) and Game development (Stroke sequence Kind of technical-tactical stroke, Bounce area Hitting area, and Stroke effectiveness)]; (b) variables to register each game [RESULTS (Games won on the set and Lost games on the set)]; (c) Variables to register each set [RESULTS (Analyzed set, Sets won, Sets lost, and Winner or loser of the analyzed set)]; (c) Variables to analyze each match [CONTEXTUAL (Gender of the players, Tournament level, Type of tournament, Tournament round, Game mode, Court surface, Laterality of the players, and Type of backhand) RESULTS (Winner or loser of the match)]

In Supplementary Data Sheet 1 you can see the definition of all variables, and all categories in detail.

## Results

The results corresponding to the design of the observational instrument after the first two phases (review of the scientific literature, pilot study, and review of the first group of experts), can be seen in Tables 2–4. All the variables related to the game were established according to the suggestions of the researchers and previous studies and were the starting point to build the structure of the instrument.

**Table 2:** List of variables and contextual categories that make up the observational instrument after the first two phases.

**VARIABLE**	**CATEGORIES**	
Gender of the players^*^	• Male •Female	• Mixed
Competition category^*^	• Senior •Junior •U-16 •U-15 •U-14 •U-13	• U-12 •U-11 •U-10 •U-9 •Others
Tournament level^**^	• Professional tournament •Semi- Professional tournament •National Tournament •Regional Tournament	• Local Tournament •Amateur Tournament •Amateur Tennis
Type of tournament^*^	• Copa Masters •Grand Slam •Premier Mandatory •Master 1,000/Premier 5 •ATP 500/Premier •ATP 250/WTA Internacional •Challengers •Futures	• ITF Circuit •National Championship •Regional Championship •Local Championship •Federated tournament •Unfederated tournament •Others
Game mode^*^	• Best of 5 sets with Tie-Break in the 5th set •Best of 5 sets without Tie-Break in the 5th set •Best of 3 sets •Two sets and Super Tie-Break if each player wins a set	• Two sets of 4 games and Super Tie-Break if each player wins a set •One Set of 4 games and Tie Break if each player wins 4 games •Others
Court surface^*^	• Hard court •Clay court •Grass court	• Indoor carpet •Others
Laterality of the players^*^	• Right handed	• Left handed
Type of backhand^*^	• One hand backhand	• Two hands backhand

* *Suggested behaviors in the review of the scientific literature (Phase 1)*,

** *Behaviors suggested by experts (Phase 1). U, Under*.

**Table 3:** List of variables and categories related with the result of the match that make up the observational instrument after the first two phases.

**VARIABLE**	**CATEGORIES**	
Winner or loser of the match^*^	• Winner of the match	• Loser of the match
Analyzed set^***^	• 1st set •2nd set •3rd set •4th set	• 5th set •Tie Break •Super Tie Break
Winner or loser of the set^***^	• Winner of the analyzed set	• Loser of the analyzed set
Games won on the set^*^	• One game won •Two games won •Three games won	• Four games won •Five games won •Six games won
Lost games on the set^*^	• One game lost •Two games lost •Three games lost	• Four games lost •Five games lost •Six games lost
Game scoreboard^**^	• 0/0 •15/0 •0/15 •15/15 •30/15 •15/30 •30/30 •40/30 •30/40 •40/40	• AD/40 •40/AD •30/0 •40/0 •0/30 •0/40 •40/15 •15/40 •Tie-break point* •Super Tie-break point^*^
Winner or loser of the point^**^	• Winner of the analyzed point	• Loser of the analyzed point

* *Suggested behaviors in the review of the scientific literature (Phase 1)*,

** *Behaviors suggested by experts (Phase 1)*,

*** *Behaviors suggested after the observational pilot test (Phase 2)*.

**Table 4:** List of variables and categories related with the game that make up the observational instrument after the first two phases.

**VARIABLE**	**CATEGORIES**	
Type of serve^*^	• 1st serve	• 2nd serve
Serve bounce area^*^	• Wide area of deuce side •Body area of deuce side •T area of deuce side •T area of advantage side •Body area of advantage side •Wide area of advantage side •Net error •Out of service line •Out of right singles sidelines on deuce side (view of receiver player)	• Out of center service line on deuce side (view of receiver player) •Out of left singles sidelines on advantage side (view of receiver player) •Out of center service line on advantage side (view of receiver player)
Serve effectiveness^*^	• Ace •Inside the service box and intercepted by the opponent	• Error
Type of stroke used by receiver player in the return, serve player after the service, receiver player after the return and penultimate and latest stroke of the point^**^	• Forehand ground stroke •Two hands backhand ground stroke •One hand backhand ground stroke •Forehand volley •Backhand volley •Smash •Forehand aproach •Two hands backhand approach •One hand backhand approach •Forehand Passing •Two hands backhand passing •One hand backhand passing •Forehand lob •Two hands backhand lob	• One hand backhand lob •Forehand drop •Two hands backhand drop •One hand backhand drop •Forehand counter drop •Two hands backhand counter drop •One hand backhand counter drop •Forehand half volley •Two hands backhand half volley •One hand backhand half volley •Others
Hitting area of the return, first stroke of serve player after the service, first stroke of the receiver player after the return and penultimate and latest stroke of the point^**^	• Behind at more than 1 m away from the baseline in the central area (+1 m) •Behind at more than 1m away from the baseline in the right area (+1 m) •Behind at more than 1 m away from the baseline baseline in the left area (+1 m) •Behind at <1 m away from the baseline in the central area (−1 m) •Behind at <1 m away from the baseline in the right area (−1 m) •Behind at <1 m away from the baseline in the left area (−1 m)	• Inside the court and behind of serve line in the central area •Inside the court and behind of serve line in the right area •Inside the court and behind of serve line in the left area •Between the service line and the net in the central area •Between the service line and the net in the right area •Between the service line and the net in the left area
Bounce area of the return, first stroke of serve player after the service, first stroke of the receiver player after the return and penultimate and latest stroke of the point** (view of the player who executes)	• The opponent hit the ball without previous bounce •Central area between net and service line •Right area between net and service line •Left area between net and service line •Central area from behind of service line until 2.74 m of baseline •Right area from behind of service line until 2.74 m of baseline	• Left area from behind of service line until 2.74 m of baseline •Central area from baseline until 2.74 m of it inside the court •Right area from baseline until 2.74 m of it inside the court •Left area from baseline until 2.74 m of it inside the court •Net error •Out of baseline •Out of right singles or dobles sideline •Out of left singles or dobles sideline
Effectiveness of return, first stroke of serve player after the service, first stroke of the receiver player after the return and penultimate and latest stroke of the point^**^	• Winner •Transition stroke	• Error

* *Suggested behaviors in the review of the scientific literature (Phase 1)*,

** *Behaviors suggested by experts (Phase 1)*.

After the first two phases, the list of variables that formed the observational instrument consisted of 38, 8 of which corresponded to contextual variables (7 suggested by the scientific literature and 1 by the experts), 7 to variables related to the result of the meeting (3 suggested by the scientific literature, 2 by the experts, and 2 included after the observational pilot study) and 23 to variables related to the game (3 suggested by the scientific literature and 20 by the experts).

In the third phase, a second group of experts (*n* = 10) made a new evaluation and there were a total of 10 modifications, 2 for new behaviors included and 8 for modifications in the existing ones. Of all of them, 1 corresponded to the variables related to the context, 4 to the results, and 5 to those that had to do with the game. The changes or modifications were made by low of Aikens's V values, and because <80% of the expert judges answered affirmatively that the variable should be included.

The main change was based on the sequence of the stroke and the kind of technical-tactical stroke. At first, the experts proposed analysing only the serve, return, serve player after the service, receiver player after the return, and penultimate and latest stroke of the point. From each of them analyzed their different types of strokes. Later on, the new expert judges proposed to analyse all the strokes, and replaced the moment of stroke variable (temporal sequence and type of stroke), by two variables: (a) the sequence variable of stroke; and (b) the kind of technical-tactical stroke, they change a single variable with many categories (moment of stroke), by two new variables with fewer categories.

In the fourth phase, after the modifications made in the previous phase, the new list was evaluated quantitatively and qualitatively by a third group of experts (*n* = 10). The Aikens's V values corresponding to said evaluation can be observed in Tables 5–7. The final list was composed of 23 variables, 8 contextual variables, 10 of result, and 5 of game (Tables 5–7). For it, it was considered that all those variables that had a value ≥ 0.81 of the Aikens's V were suitable to be part of the instrument (Table 8). In all cases, 100% of the expert judges answered affirmatively that the variable should be included.

**Table 5:** Final list of variables and contextual categories that make up the observational instrument.

**VARIABLE**	**CATEGORIES**	
Gender of the players^*^	• Male •Female	• Mixed
Tournament level^*^	• Professional tournament •Semi- Professional tournament •National Tournament •Regional Tournament	• Local Tournament •Amateur Tournament •Amateur Tennis
Type of tournament^*^	• Copa Masters •Grand Slam •Premier Mandatory •Master 1,000/Premier 5 •ATP 500/Premier •ATP 250/WTA Internacional •Challengers •Futures	• ITF Circuit •National Championship •Regional Championship •Local Championship •Federated tournament •Unfederated tournament •Others
Tournament round^**^	• Round robin •Final •Semifinal •Quarter finals •Best sixteen	• Best 32 •Best 64 •Best 128 •Others
Game mode^*^	• Best of 5 sets with Tie-Break in the 5th set •Best of 5 sets without Tie-Break in the 5th set •Best of 3 sets •Two sets and Super Tie-Break if each of the players wins a set	• Two sets of 4 games and Super Tie-Break if each of the players wins a set •One Set of 4 games and Tie Break if each of the players wins 4 games •Others
Court surface^*^	• Hard court •Clay court •Grass court	• Indoor carpet •Others
Laterality of the players^*^	• Right handed	• Left handed
Type of backhand^*^	• One hand backhand	• Two hands backhand

* *Behaviors selected after the first and second phase*,

** *Behaviors suggested by experts (Phase 3 and 4)*.

**Table 6:** Final list of variables and categories related with the result of the match that make up the observational instrument.

**VARIABLE**	**CATEGORIES**	
Winner or loser of the match^*^	• Winner	• Loser
Analyzed set^**^	• 1st set •2nd set •3rd set •4th set	• 5th set •Tie Break •Super Tie Break
Sets won^**^	• One set won	• Two sets won
Sets lost^**^	• One set lost	• Two sets lost
Winner or loser of the analysed set^**^	• Winner of the set	• Loser of the set
Games won on the set^*^	• One game won •Two games won •Three games won	• Four games won •Five games won •Six games won
Lost games on the set^*^	• One game lost •Two games lost •Three games lost	• Four games lost •Five games lost •Six games lost
Winner or loser of the game ^**^	• Winner of the game	• Loser of the game
Game score^*^	• 0/0 •15/0 •0/15 •15/15 •30/15 •15/30 •30/30 •40/30 •30/40 •40/40	• AD/40 •40/AD •30/0 •40/0 •0/30 •0/40 •40/15 •15/40 •Tie-break point* •Super Tie-break point^*^
Winner or loser of the analyzed point^*^	• Winner of the point	• Loser of the point

* *Behaviors selected after the first and second phase*,

** *Behaviors suggested by experts (Phase 3 and 4)*.

**Table 7:** Final list of variables and categories related with the development of the game that make up the observational instrument.

**VARIABLE**	**CATEGORIES**	
Stroke sequence^**^	• Serve •Return •3rd stroke of the point	• 4th stroke of the point, 5th stroke of the point… •Penultimate stroke of the point •Last stroke of the point
Kind of technical and tactical stroke^**^	*Category of basic strokes:* •1st serve •2nd serve •Forehand ground stroke •Two hands backhand ground stroke	• One hand backhand ground stroke •Forehand volley •Backhand volley •Smash
	Category of especial strokes: •Forehand lob •Two hands backhand lob •One hand backhand lob •Forehand lob return •Two hands backhand lob return •One hand backhand lob return •Forehand drop	• Two hands backhand drop •One hand backhand drop •Forehand half volley •Two hands backhand half volley •One hand backhand half volley
	Category of situation strokes: •Forehand approach Two hands backhand approach One hand backhand approach •Forehand counter drop •Two hands backhand counter drop •One hand backhand counter drop Forehand Passing	Two hands backhand passingOne hand backhand passing•Forehand return •Two hands backhand return •One hand backhand return •Forehand drop return •Two hands backhand drop return •One hand backhand drop return •Forehand return approach •Two hands backhand return approach •One hand backhand return approach •Forehand passing of return •Two hands backhand passing of return •One hand backhand passing of return •Others
Bounce area ^**^	Category of bounce area for the serve*: •Wide area of deuce side •Body area of deuce side •T area of deuce side •T area of advantage side •Body area of advantage side •Wide area of advantage side •Net error	• Out of service line •Out of right singles sidelines on deuce side (view of receiver player) •Out of center service line on deuce side (view of receiver player) •Out of left singles sidelines on advantage side (view of receiver player) •Out of center service line on advantage side (view of receiver player)
	Category of bounce area for return, third stroke, fourth stroke penultimate and last stroke **: The opponent hit the ball without previous bounce Central area between net and service line Right area between net and service line Left area between net and service line Central area from behind of service line until 2.74 m of baseline •Right area from behind of service line until 2.74 m of baseline	• Left area from behind of service line until 2,74 m of baseline Central area from baseline until 2.74 m of it inside the courtRight area from baseline until 2.74 m of it inside the courtLeft area from baseline until 2.74 m of it inside the court•Net error •Out of baseline •Out of right singles sideline •Out of left singles sideline
Hitting area**(view of player who executes the stroke)	• Behind from the baseline in the central area •Behind from the baseline in the right area •Behind from the baseline in the left area •Inside the court and behind of serve line in the central area	• Inside the court and behind of serve line in the right area •Inside the court and behind of serve line in the left area •Between the service line and the net in the central area •Between the service line and the net in the right area •Between the service line and the net in the left area
Stroke effectiveness^**^	• Ace •Winner •Transition stroke^**^	• Previous stroke of an opponent error** •Error

^*^Behaviours selected after the first and second phase,

** *Behaviors suggested by experts (Phase 3 and 4)*.

**Table 8:** Values of pertinence, definition (Aiken's V) and reliability (Cohen's kappa) of definitive variables and categories of the observational instrument.

**Variables**	**Pertinence (V Aiken)**	**Definition (V Aiken)**	**Reliability Inter-Observer (Cohen's kappa)**	**Reliability Intra-Observer (Cohen's kappa)**
**CONTEXTUAL**				
Gender of the players	1	1	1	1
Tournament level	0.97	0.97	1	1
Type of tournament	0.96	0.96	1	1
Tournament round	0.96	0.96	1	1
Game mode	0.99	0.99	1	1
Court surface	0.97	0.97	1	1
Laterality of the players	0.99	0.99	1	1
Type of backhand	0.98	0.98	1	1
**RESULT**				
Winner or loser of the match	0.98	0.95	
Analyzed set	0.94	0.94	1	1
Sets won	0.99	0.94	1	1
Sets lost	1	0.96	1	1
Winner or loser of the analyzed set	0.99	0.97	1	1
Games won on the set	0.99	0.97	1	1
Lost games on the set	0.99	0.97	1	1
Game scoreboard	1	0.95	1	1
Winner or loser of the analyzed point	0.99	0.99	1	1
**GAME DEVELOPMENT**				
Stroke sequence			1	1
Kind of technico-tactical stroke	0.97	0.95	0.9	1
Bounce area	0.96	0.94	0.86	0.9
Hitting area	0.97	0.95	0.81	0.9
Stroke effectiveness	0.96	0.97	1	1

The data from the fifth phase showed high reliability values, as it can be seen in Table 8, the lowest value was found in the stroking zone variable (0.81).

## Discussion

The study carried out shows all the phases that have been necessary to design, validate, and test the confidence of the observational instrument that analyses the technical-tactical actions in the singles tennis. The procedure has required an updated review of the literature, a pilot study, the training of the observers, and the participation of a large number of experts (Villarejo et al., 2014; Anguera and Hernández-Mendo, 2015; Serra-Olivares and García-López, 2016). The procedure followed has been very similar to that used by Villarejo et al. (2014) in rugby, Palao et al. (2015) for beach volleyball, although it differs from that of Gorospe et al. (2005); James et al. (2005), or Jones et al. (2008), mainly due to the type of participation of the experts and the pilot study.

The pilot study allowed defining, specifying, and adapting the initial list of behaviors to the real competition situation (Anguera, 2003; Anguera and Hernández-Mendo, 2015). Later on, the first observation allowed to verify the frequency of appearance of the behaviors proposed by the first group of experts, to eliminate or include in other categories those that showed little frequency of occurrence and incorporate those observed that were not initially included.

The experts have helped to significantly improve the instrument through: (a) inclusion of new specific behaviors of the game; and (b) improve and clarify the definitions of the variables and their relevance to the different categories (Mills et al., 2012). Both contributions at a qualitative level have definitely been decisive in designing and validating the instrument. It has gone from 38 initial variables of the provisional list designed by the researchers, experts and observer (8 contextual, 7 of result, and 23 of game) to 23 final variables (8 contextual, 10 of result, and 5 of the game). On the other hand, the observers have also played an important role, since once their training process has ended, they have helped to specify the criteria by which the different categories are distinguished and their contribution has simplified the registration instrument.

Different groups of experts have been used in the design and validation of the instrument. A total number of four observers and 23 experts have participated. The number of expert judges, despite being a specific observation instrument for a single sport, is much higher than those used in similar studies (Villarejo et al., 2014; García et al., 2016; Chacón-Moscoso et al., 2018; Gamonales et al., 2018). These high values provide a high consistency in the content validity of the observation instrument. In this sense, the high qualification of the different expert judges stands out, following all of them the three criteria of inclusion: graduates in Sports Sciences and Sport, with federated degree as coaches and with more than 10 years of experience as trainers. This high training has allowed them to provide theoretical, but especially practical knowledge, of their sport experiences. Their quantitative and qualitative contributions have been the basis for the design of this instrument.

Finally, with respect to the expert judges, it is remarkable that the different expert judges have been participating in different phases, without any expert judge repeating in any of the phases. So it could be noted that there have been three panels of expert judges. The first panel of expert judges was formed by the first three experts who initially designed the instrument, the second panel was composed of 10 other experts who contributed the first modifications; and finally, a third panel formed by 10 other experts who have ratified the previous proposals. During the whole process there has been no communication between the different panels, but they have been acting one after another (in cascade process). This has allowed them to act with total independence (Escobar-Pérez and Cuervo-Martínez, 2008; Kimberlin and interstein, 2008; Drost, 2011).

At the statistical level, it is demonstrated that the instrument is prepared to measure the technical-tactical behaviors in singles tennis, and the Aiken's V values show a positive evaluation of the content of the different items (Gómez et al., 2014; Zartha et al., 2018). The values of the quantitative evaluation contributed by the third group of experts were superior in all cases to the value of 0.81, so no new modification had to be made. The fact that a large number of experts have participated in the design of the observation instrument, together with the statistical treatment and the pilot study, has minimized the subjective opinion of the coaches on how they understand the game.

The possibility of establishing a link between previous and subsequent actions (Reid et al., 2016), as in the case of the return, serve player after the service, receiver player after the return and penultimate and latest stroke of the point, allows knowing how the game is conditioned and affects the result of the point.

Observers training increased the effectiveness of the observation and improved the coding criteria. The level of agreement between observers (inter and intra observer), allowed to affirm that the observation carried out is reliable (Liu et al., 2013). The observation manual carried out helped the observers, to acquire the necessary skills to carry out the observation (Losada and Manolov, 2015).

The design of the instrument has some limitations, as it only analyses the position of the player who is in the hitting phase, but not the one of the player who is in the waiting phase. This information could be useful and influence the decision of the player who is about to impact the ball, since as Lebed (2006) states, the behaviors in sport are influenced by an infinite number of factors. Due to the complexity of this system, it is difficult to apply collection information systems with the aim of assessing players' performance in competition (Villarejo et al., 2014). Besides, the instrument does not record data about the game or rest times or about physical components, such as the number or direction of the displacements. However, and under our knowledge, this tool will greatly facilitate the work of researchers and coaches, becoming a valid instrument to assess technical-tactical actions in a sport such as singles tennis.

## Conclusions

Therefore, the instrument designed is valid to analyse from a technical-tactical perspective the service, return, strokes in the middle of rally, penultimate and latest stroke of the point. In this way it is possible to check the possible relationships between them and with respect to the result of the point, thus assessing the differences that may exist between winners and losers.

## Practical applications

This information can be used by players and coaches to evaluate their own actions and their opponents from a technical-tactical perspective. This would help to increase performance through: (a) the improvement of training programs aimed at the specific improvement of technical-tactical skills; and (b) the analysis of the technical-tactical qualities of the rivals.

## Ethics statement

This study respected the ethical principles established by the UNESCO Declaration on Bioethics and Human Rights. The parents or guardians of the players were informed of the study and gave their written consent in accordance with the Declaration of Helsinki. The study was approved by the Ethics Committee of University of Murcia (Spain) with ID 1925/2018.

## Author contributions

GT-L, and EO-T contributed with the conception and design of the study. AF-G and DC-M organized the database. JG-E and EO-T performed the statistical analysis. EO-T, GT-L, AF-G wrote the first draft of the manuscript. JG-E, DC-M, GT-L, and EO-T wrote sections of the manuscript. All the authors contributed to the revisión of the manuscript, and read and approved the presented version.

### Conflict of interest statement

The authors declare that the research was conducted in the absence of any commercial or financial relationships that could be construed as a potential conflict of interest.
