# Supplementary material for: Design and Validation of an Observational Instrument for the Technical-Tactical Actions in Singles Tennis
Source: Front Psychol. 2018 Dec 3;9:2418. doi: 10.3389/fpsyg.2018.02418 (PMC6287015; doi:10.3389/fpsyg.2018.02418)
Supplement: Supplementary file 1 [file Data_Sheet_1.docx]

Supplementary Data Sheet 1: Observation instrument for Individual Tennis (IOTInd)

**OBSERVATION INSTRUMENT FOR SINGLES TENNIS (OITSin)**

The present instrument of evaluation measures the technical and tactical actions executed in a singles tennis match.

The unit of measurement is the "Stroke". Each time that a player hits the ball, a total of 23 variables will be recorded. All these variables have been divided in three groups to be registered easily:

- Contextual variable
- Variables related with the result of the match
- Variables related with the development of play

***1. CONTEXTUAL VARIABLES***

The first section of the instrument is made up of by the variables related to the context in which the match is played. These variables are defined for the same match, so all the data will be repeated constantly throughout it.

- 1. Gender of the players (Gen): The gender of the players is specified.

| 1 | Male |
| --- | --- |
| 2 | Female |

- 1. Tournament Level (Lev): Depending on the degree of professionalism of the tournament, several levels are established.

| 1 | Professional |
| --- | --- |
| 2 | Semi-Professional |
| 3 | National |
| 4 | Regional |
| 5 | Local |
| 6 | Amateur |
| 7 | Others |

1. *Proffesional: ATP, WTA e ITF tournament (U-18 and U-16).*
2. *Semi-Profesional: Futures and Challengers tournaments.*
3. *National: National tournaments of any category.*
4. *Regional: Regional tournaments of any category.*
5. *Local: Local tournaments of any category.*
6. *Tenis Amateur: Local federated (except local, regional or national) or unfederated tournaments of any category.*
7. *Others: any other competition that is not included inside of the previous ones.*
   1. Type of tournament (Tou): Inside of each different degrees of professionalism of the tournament, there are different levels. In the case of professional tournaments, the level is directly related to the money that is distributed in prizes.

| 1 | Masters Cup |
| --- | --- |
| 2 | Grand Slam |
| 3 | Premier Mandatory |
| 4 | Master 1000 / Premier 5 |
| 5 | ATP 500 / Premier |
| 6 | ATP 250 / WTA Internacional |
| 7 | Challengers |
| 8 | Futures |
| 9 | ITF Circuit |
| 10 | National Championship |
| 11 | Regional Championship |
| 12 | Local Championship |
| 13 | Federated Tournaments |
| 14 | Unfederated Tournaments |
| 15 | Others: any other competition that is not included inside of the previous ones |

- 1. Tournament Round (Tou_Rou): It will be specified in which phase of the tournament the analyzed match is.

| 0 | Round Robin |
| --- | --- |
| 1 | Final |
| 2 | Semifinal |
| 4 | Quarter final |
| 8 | Best sixteen |
| 16 | Best thirty two |
| 32 | Best sixty four |
| 64 | Best one hundred twenty-eight |
| 100 | Others round previous to best one hundred twenty-eight |

- 1. Game Mode (Gam_Mod): It will be specified the number and kind of sets that match is played.

| 1 | Best of 5 sets with Tie-Break in the 5th set |
| --- | --- |
| 2 | Best of 5 sets without Tie-Break in the 5th set |
| 3 | Best de 3 sets |
| 4 | Two sets and Super Tie-Break if each player wins a set |
| 5 | Two sets of 4 games and Super Tie-Break if each player wins a set |
| 6 | One set of 4 games and Tie Break if each player wins 4 games |
| 7 | Others |

- 1. Court Surface (Cou_Sur): It will be recorded the Surface in wich the match is played.

| 1 | Hard court |
| --- | --- |
| 2 | Clay court |
| 3 | Grass court |
| 4 | Indoor carpet |
| 5 | Others |

- 1. Laterality of the palyers (Lat_Pla): The player's dominant arm is registered.

| 1 | Right handed |
| --- | --- |
| 2 | Left handed |

- 1. Type of Backhand (Typ_Bac): It will be recorded if the players grip de racket with one or two hands for hit the ball with her/his backhand.

| 1 | One hand backhand |
| --- | --- |
| 2 | Two hands backhand |

***2. VARIABLES RELATED WITH THE RESULT***

The result variables provide information about the score of the match in its different phases.

- 1. Winner or loser of the match (Win_Los_Mat): It will be registerred if the analyzed player is the winner or the loser of the match.

| 1 | Winner |
| --- | --- |
| 2 | Loser |

- 1. Analyzed set (Set): Number of set that is being analyzed in the match.

| 1 | 1st Set |
| --- | --- |
| 2 | 2nd Set |
| 3 | 3rd Set |
| 4 | 4th Set |
| 5 | 5th Set |
| 6 | Tie Break |
| 7 | Super Tie Break |

- 1. Sets won (Sets_Won): Number of sets that the player has won at the moment of the analysis.

| 1 | One set won |
| --- | --- |
| 2 | Two sets won |

- 1. Sets lost (Sets_Lost): Number of sets that the player has lost at the moment of the analysis

| 1 | One set lost |
| --- | --- |
| 2 | Two set lost |

- 1. Winner or loser of the analysed set (Win_Los_Set): It will be registered if the player who hit the ball is the winner or loser of the analysed set.

| 1 | Winner of the set |
| --- | --- |
| 2 | Loser of the set |

- 1. Games won on the set (Gam_Won): Number of games that the player has won in the set at the moment of the analysis.

| 1 | One game won |
| --- | --- |
| 2 | Two games won |
| 3 | Three games won |
| 4 | Four games won |
| 5 | Five games won |
| 6 | Six games won |

- 1. Games lost on the set (Gam_Lost): Number of games that the player has lost in the set at the moment of the analysis.

| 1 | One game lost |
| --- | --- |
| 2 | Two games lost |
| 3 | Three games lost |
| 4 | Four games lost |
| 5 | Five games lost |
| 6 | Six games lost |

- 1. Winner or loser of the game (Win_Los_Gam): It will be recorded if the player who hit the ball is the winner or loser of the analysed game.

| 1 | Winner of the game |
| --- | --- |
| 2 | Loser of the game |

- 1. Game score (Gam_Sco): The result of the game in the moment of the analyzed point it will be noted.

| 00 | 0/0 |
| --- | --- |
| 10 | 15/0 |
| 01 | 0/15 |
| 11 | 15/15 |
| 21 | 30/15 |
| 12 | 15/30 |
| 22 | 30/30 |
| 32 | 40/30 |
| 23 | 30/40 |
| 33 | 40/40 |
| 43 | AD/40 |
| 34 | 40/AD |
| 20 | 30/0 |
| 30 | 40/0 |
| 02 | 0/30 |
| 03 | 0/40 |
| 31 | 40/15 |
| 13 | 15/40 |
| 7 | Tie-Break points* |
| 10 | Super Tie-Break points * |

**For the score in the Tie-Breaks, it will be registered the result preceded by a 7 (example: 2-1 for the winner of the match: the code would be 721). For the Super Tie-Break, it is the same with the numer 10 instead of 7.*

- 1. Winner or loser of the analysed point (Win_Los_Pt): It will be registered if the player who hit the ball win or lose the analysed point.

| 1 | Winner of the point |
| --- | --- |
| 2 | Loser of the point |

***3. VARIABLES RELATED WITH THE RESULT OF THE MATCH***

The game variables record different technical and tactical dimensions that occur from the beginning of the point when the ball is hit by the server, until the point ends.

- 1. Variables related with the sroke sequence (Sec_Str): Corresponds to the temporal sequence of strokes that happens in the development of a point.

| 1 | Serve |
| --- | --- |
| 2 | Return |
| 3 | 3rd stroke of the point |
| 4 | 4th stroke of the point golpe |
| 5 | 5th stroke of the point golpe |
| … | …. |
| 01 | Penultimate stroke of the point golpe |
| 00 | Last stroke of the point golpe |

*1: Stroke that corresponds to the serve modality.*

*2: Stroke that corresponds to the return modality*

*3, 4, 5, 6….: Sequence of strokes ordered sequentially that occurs after the rest and that ends before the penultimate stroke of the point. An example would be: a point that has 8 strokes between players would be coded serve, return, third stroke, fourth stroke, Fifth stroke, sixth stroke, penultimate stroke and last stroke).*

*01: Stroke that occurs before the last stroke made in the point between two players*

*00: Last stroke that occurs in the dispute of a point between two players*

- 1. Variables related with the technical and tactical dimension of the analyzed stroke (Tec_Tac_St): The technical and tactical mode of stroke is recorded, using the criteria of the Royal Spanish Tennis Federation (RFET). Although all strokes are exclusive, to simplify the record they have been divided into three groups: Basic strokes, Especial strokes and Situation shots.

| 1 | 1st serve |
| --- | --- |
| 2 | 2nd serve |
| 3 | Forehand return |
| 4 | Two hands backhand return |
| 5 | One hand backhand return |
| 6 | Forehand ground stroke |
| 7 | Two hands backhand ground stroke |
| 8 | One hand backhand ground stroke |
| 9 | Forehand volley |
| 10 | Backhand volley |
| 11 | Smash |
| 12 | Forehand lob |
| 13 | Two hands backhand lob |
| 14 | One hand backhand lob |
| 15 | Forehand lob return |
| 16 | Two hands backhand lob return |
| 17 | One hand backhand lob return |
| 18 | Forehand drop |
| 19 | Two hands backhand drop |
| 20 | One hand backhand drop |
| 21 | Forehand drop return |
| 22 | Two hands backhand drop return |
| 23 | One hand backhand drop return |
| 24 | Forehand half volley |
| 25 | Two hands backhand half volley |
| 26 | One hand backhand half volley |
| 27 | - Forehand approach |
| 28 | - Two hands backhand approach |
| 29 | - One hand backhand approach |
| 30 | Forehand return approach |
| 31 | Two hands backhand return approach |
| 32 | One hand backhand return approach |
| 33 | - Forehand counter drop |
| 34 | - Two hands backhand counter drop |
| 35 | - One hand backhand counter drop |
| 36 | Forehand Passing |
| 37 | Two hands backhand passing |
| 38 | One hand backhand passing |
| 39 | Forehand passing of return |
| 40 | Two hands backhand passing of return |
| 41 | One hand backhand passing of return |
| 42 | Others |

Basic Strokes:

*1 First Serve: Stroke by which the ball is put into play during a point. It must be hit behind the baseline and between the extensions of the singles sideline (right of left, depends if the player serve in deuce or advantage side) and central service line. Always it executed without a previous bounce (in the air), and it can be hit above or below the height of the head. It is the first attempt that the player has to put the ball in play when he/she is at the serve.*

*2 Second Serve: Stroke by which the ball is put into play during a point. It must be hit behind the baseline and between the extensions of the singles sideline (right of left, depends if the player serve in deuce or advantage side) and central service line. Always it executed without a previous bounce (in the air), and it can be hit above or below the height of the head. It is the second attempt that the player has to put the ball in play when he/she is at the serve.*

*6 Forehand ground stroke: Stroke that takes place after the ball's bounce and that is executed by the dominant side of the player (right side for right-handed and left for left-handed). It is generally considered as a ground stroke, although it can be executed in any area of the court.*

*7 Two hands backhand ground stroke: Stroke that is carried out after the ball's bounce and is executed by the non-dominant side of the player (left side for right-handed and right for left-handed) holding the grip with two hands. It is generally considered as a ground stroke, although it can be executed in any area of the court.*

*8 One hand backhand ground stroke: Stroke that is carried out after the ball's bounce and is executed by the non-dominant side of the player (left side for right-handed and right for left-handed) holding the grip with one hand. It is generally considered as a ground stroke, although it can be executed in any area of the court.*

*9 Forehand volley: Stroke that is made before the bounce of the ball as a response to a stroke from the opponent. The impact zone does not usually exceed the height of the player's head and is executed by the dominant side of the player (right side for right-handed and left for left-handed). It is generally considered as a net stroke, although it can be executed in any area of the court as long as it is executed without a previous bounce of the ball.*

*10 Backhand volley: Stroke that is made before the bounce of the ball as a response to a stroke from the opponent. The impact zone does not usually exceed the height of the player's head and is executed by the dominant side of the player (left side for right-handed and right for left-handed). It is generally considered as a net stroke, although it can be executed in any area of the court as long as it is executed without a previous bounce of the ball.*

### 11 Smash: Stroke that is made hitting the ball over the head. It is similar in execution to service, but with the difference that the ball comes from the opponent. It can be done before or after the bounce of the ball. It is generally considered as a net stroke, although it can be executed in any area of the court.

### Especials strokes:

*12 Forehand lob: Forehand stroke that always discribe an elliptical trajectory. Usually it is carried out when the player is in defensive phase in order to gain time to recover the position or surpass his opponent located near the net*

*13 Two hands backhand lob: Two hands backhand stroke that always discribe an elliptical trajectory. Usually it is carried out when the player is in defensive phase in order to gain time to recover the position or surpass his opponent located near the net*

*14 One hand backhand lob: One hand backhand stroke that always discribe an elliptical trajectory. Usually it is carried out when the player is in defensive phase in order to gain time to recover the position or surpass his opponent located near the net.*

*18 Forehand drop: Forehand stroke whose intention is to leave the ball as close as possible to the net with the intention of directly winning the point or attract the opponent to the net.*

*19 Two hands backhand drop: Two hands backhand stroke whose intention is to leave the ball as close as possible to the net with the intention of directly winning the point or attract the opponent to the net.*

*20 One hand backhand drop: One hand backhand stroke whose intention is to leave the ball as close as possible to the net with the intention of directly winning the point or attract the opponent to the net.*

*24 Forehand half volley: Forehand stoke in which the impact it is produced just after the ball bounce when it is in the ascendant phase, so usually the height of it is between the ground and the hip.*

*25 Two hands backhand half volley: Two hands backhand stoke in which the impact it is produced just after the ball bounce when it is in the ascendant phase, so usually the height of it is between the ground and the hip.*

*26 One hand backhand half volley: One hand backhand stoke in which the impact it is produced just after the ball bounce when it is in the ascendant phase, so usually the height of it is between the ground and the hip.*

### Situation Strokes:

*3 Forehand return: Stroke that is executed after the bounce of the opponent service and that is executed by the dominant side of the player (right side for right-handed and left for left-handed). It is generally considered as a ground stroke, although it can be executed in any area of the court.*

*4 Two hands backhand return: Stroke that is executed after the opponent service and that is executed by the no dominant side of the player (left side for right-handed and left for left-handed) gripping the racket with two hands. It is generally considered as a ground stroke, although it can be executed in any area of the court.*

*5 One hand backhand return: Stroke that is executed after the opponent service and that is executed by the no dominant side of the player (left side for right-handed and left for left-handed) gripping the racket with one hand. It is generally considered as a ground stroke, although it can be executed in any area of the court.*

*15 Forehand lob return: Forehand stroke that the receiver player executed after the serve of his/her opponent that always discribe an elliptical trajectory. Normally it is carried out when the player is in defensive phase in order to gain time to recover the position or surpass his opponent that comming at the net after serve.*

*16 Two hands backhand lob return: Two hands backhand stroke that the receiver player executed after the serve of his/her opponent that always discribe an elliptical trajectory. Normally it is carried out when the player is in defensive phase in order to gain time to recover the position or surpass his opponent that comming at the net after serve.*

*17 One hand backhand lob return: One hand backhand stroke that the receiver player executed after the serve of his/her opponent that always discribe an elliptical trajectory. Normally it is carried out when the player is in defensive phase in order to gain time to recover the position or surpass his opponent that comming at the net after serve.*

*21 Forehand drop return: Forehand stroke that the receiver player executed after the serve of his/her opponent whose intention is to leave the ball as close as possible to the net with the intention of directly winning the point or attract the opponent to the net.*

*22 Two hands backhand drop return: Two hands backhand stroke that the receiver player executed after the serve of his/her opponent whose intention is to leave the ball as close as possible to the net with the intention of directly winning the point or attract the opponent to the net*

*23 One hand backhand drop return: One hand backhand stroke that the receiver player executed after the serve of his/her opponent whose intention is to leave the ball as close as possible to the net with the intention of directly winning the point or attract the opponent to the net*

*27 Forehand approach: Forehand stroke that precedes the player's movement towards the net and is executed after the ball bounce.*

*28 Two hands backhand approach: Two hands backhand stroke that precedes the player's movement towards the net and is executed after the ball bounce.*

*29 One hand backhand approach: One hand backhand stroke that precedes the player's movement towards the net and is executed after the ball bounce.*

*30 Forehand approach return: Forehand stroke that happens after the opponent's serve that precedes the player's movement towards the net and is executed after the ball is bounced.*

*31 Two hands backhand approach return: Two hands backhand stroke that happens after the opponent's serve that precedes the player's movement towards the net and is executed after the ball is bounced.*

*32 One hand backhand approach return: One hand backhand stroke that happens after the opponent's serve that precedes the player's movement towards the net and is executed after the ball is bounced.*

*33 Forehand counter drop: Forehand stroke that is executed after a drop of the opponent, with the aim of leaving the ball as close as possible to the net with the intention of directly winning the point or attracting the opponent to the network*

*34 Two hands backhand counter drop: Two hands backhand stroke that is executed after a drop of the opponent, with the aim of leaving the ball as close as possible to the net with the intention of directly winning the point or attracting the opponent to the network*

*35 One hand backhand counter drop: One hand backhand stroke that is executed after a drop of the opponent, with the aim of leaving the ball as close as possible to the net with the intention of directly winning the point or attracting the opponent to the network*

*36 Forehand passing-shot: Forehand stroke that is made after the rise of the opponent to the net and usually it when the player is in defensive phase. It is always produced with a previous bounce of the ball, it has a low trajectory over the net and the aim of overcoming directly the opponent placed in the net or forces him to make a stroke of great difficulty.*

*37 Two hands backhand passing-shot: Two hands backhand that is made after the rise of the opponent to the net and usually it when the player is in defensive phase. It is always produced with a previous bounce of the ball, it has a low trajectory over the net and the aim of overcoming directly the opponent placed in the net or forces him to make a stroke of great difficulty.*

*38 One hand backhand passing-shot: One hand backhand that is made after the rise of the opponent to the net and usually it when the player is in defensive phase. It is always produced with a previous bounce of the ball, it has a low trajectory over the net and the aim of overcoming directly the opponent placed in the net or forces him to make a stroke of great difficulty.*

*39 Forehand passing-shot return: Forehand stroke next to the opponent's serve that is made after the rise of the service player to the net and usually it carried out when the player is in defensive phase. It is always produced with a previous bounce of the ball, it has a low trajectory over the net and the aim of overcoming directly the opponent placed in the net or forces him to make a stroke of great difficulty.*

*40 Two hands backhand passing-shot return: Two hands backhand stroke next to the opponent's serve that is made after the rise of the service player to the net and usually it carried out when the player is in defensive phase. It is always produced with a previous bounce of the ball, it has a low trajectory over the net and the aim of overcoming directly the opponent placed in the net or forces him to make a stroke of great difficulty.*

*41 One hand backhand passing-shot return: One hand backhand stroke next to the opponent's serve that is made after the rise of the service player to the net and usually it carried out when the player is in defensive phase. It is always produced with a previous bounce of the ball, it has a low trajectory over the net and the aim of overcoming directly the opponent placed in the net or forces him to make a stroke of great difficulty.*

*42 Others: regarding modalities that do not appear in the list.*

3.3 Variables related with the bounce area of the ball: Area of the court in where the ball bounce after the stroke of a player. Although all areas are exclusive, to simplify the record they have been divided into two groups: serve area and return, third stroke, fourth stroke… penultimate and last stroke of the point.

Bounce area of the serve (Ser_Bou) (FIGURA 1): Corresponds to the area of the service box in which the ball bounces after the serve. Each service box side will be divided into 3 excluding areas of the same size (6.40 x 1.37 meters) and also contemplating depth, laterals and net errors.

| 1 | Wide area of deuce side |
| --- | --- |
| 2 | Body area of deuce side |
| 3 | T area of deuce side |
| 4 | T area of advantage side |
| 5 | Body area of advantage side |
| 6 | Wide area of advantage side |
| 7 | Net error |
| 8 | Out of service line |
| 9 | Out of right singles sideline on deuce side (view of receiver player) |
| 10 | Out of center service line on deuce side (view of receiver player) |
| 11 | Out of left singles sideline on advantage side (view of receiver player) |
| 12 | Out of center service line on advantage side (view of receiver player) |

*1: Serve directed to the deuce service box and bouncing inside the box and in the area between the right singles sideline (seen from the position of the return player) and an imaginary line located at 1.37 meters from it.*

*2: Serve directed to the deuce service box and bouncing inside the box and in the area between the imaginary line located at 1.37 meters from the right singles sideline (seen from the position of the return player) and an imaginary line located at 1.37 meters from the center service line.*

*3: Serve directed to the deuce service box and bouncing inside the box and in the area between the imaginary line located at 1.37 meters from the center service line (seen from the position of the return player) that divides both service boxes and this same line.*

*4: Serve directed to the advantage service box and bouncing inside the box and in the area between the imaginary line located at 1.37 meters from the center service line (seen from the position of the return player) that divides both service boxes and this same line.*

*5: Serve directed to the advantage service box and bouncing inside the box and in the area between the imaginary line located at 1.37 meters from the left singles sideline (seen from the position of the return player) and an imaginary line located at 1.37 meters from the center service line*

*6: Serve directed to the advantage service box and bouncing inside the box and in the area between the left singles sideline (seen from the position of the return player) and an imaginary line located at 1.37 meters from it.*

*7: The ball impact in the net and does not pass into the opponent court.*

*8: The ball bounces out and behind the service line*

*9: The ball bounces out of right sideline on deuce side, inside the right tram (seen from the position of the return player) or farther away from it*

*10. The ball bounces out of center service line on deuce side, inside the advantage service box or farther away from it*

*11. The ball bounces out of left sideline on deuce side, inside the left tram (seen from the position of the return player) or farther away from it*

*12. The ball bounces out of center service line on advantage side, inside the deuce service box or farther away from it*

*** *The codes from 7 to 12 will only be used in the event that the player commit service fault to specify the kind of error.*

| 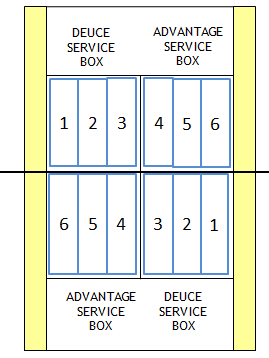 | 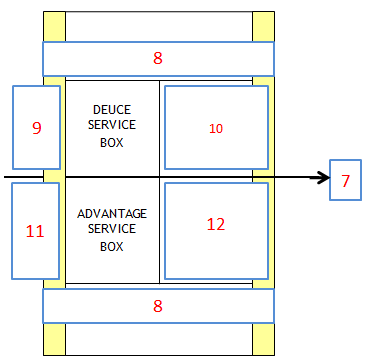 |
| --- | --- |

Figure 1. Bounce area of service *(the numbers in red color correspond to the áreas consider like errors)*

- - 1. Bounce area for return, third stroke, fourth stroke… penultimate and last stroke (Bou_Ar) (Figura 2): Corresponds to the area of the court in which the ball bounces after the player hit the ball in the return, third, fourth… and penultimate and last stroke of the point.

| 0 | The opponent hit the ball without previous bounce |
| --- | --- |
| 11 | Central area between net and service line |
| 12 | Right area between net and service line |
| 13 | Left area between net and service line |
| 21 | Central area from behind of service line until 2,74m of baseline |
| 22 | Right area from behind of service line until 2,74m of baseline |
| 23 | Left area from behind of service line until 2,74m of baseline |
| 31 | Central area from baseline until 2,74m of it inside the court |
| 32 | Right area from baseline until 2,74m of it inside the court |
| 33 | Left area from baseline until 2,74m of it inside the court |
| 7 | Net error |
| 8 | Out of baseline |
| 9 | Out of right singles sideline |
| 10 | Out of left singles sideline |

*11: The ball bounces in the area between the net and the service line and always at a minimun distance of 2.74 meters with respect to the singles sidelines.*

*12: The ball bounces in the area between the net and the service line and always from the right singles sideline (perspective of the player who is going to hit) of the individual court up to a maximum distance of 2.74 meters from it.*

*13: The ball bounces in the area between the net and the service line and always from the left singles sideline (perspective of the player who is going to hit) of the individual court up to a maximum distance of 2.74 meters from it.*

*21: The ball bounces in the area that goes from the service line to the imaginary line parallel to it and which is at 2.74 meters. In addition, the bounce of the ball must be at a minimum distance of 2.14 meters with respect to the singles sidelines*

*22: The ball bounces in the area that goes from the service line to the imaginary line parallel to it and which is at 2.74 meters. In addition, the bounce of the ball must always be placed from the right singles sideline (perspective of the player who is going to hit) to a maximum distance of 2.74 meters from it.*

*23: The ball bounces in the area that goes from the service line to the imaginary line parallel to it and which is at 2.74 meters. In addition, the bounce of the ball must always be placed from the left singles sideline (perspective of the player who is going to hit) to a maximum distance of 2.74 meters from it.*

*31: The ball bounces in the area that extends from the baseline to an imaginary line parallel to it and which is at 2.74 meters inside the individual court. In addition, the bounce of the ball must always be at a minimum distance of 2.14 meters with respect to the singles sidelines*

*32: The ball bounces in the area that extends from the baseline to an imaginary line parallel to it and wich is at a 2.74 meters inside the individual court. In addition, the bounce of the ball must be at a maximum distance of 2.14 meters from the right of sinles sideline (perspective of the player who is going to hit)*

*33: The ball bounces in the area that extends from the baseline to an imaginary line parallel to it and wich is at a 2.74 meters inside the individual court. In addition, the bounce of the ball must be at a maximum distance of 2.14 meters from the left of sinles sideline (perspective of the player who is going to hit)*

*7: The ball impact in the net and does not pass into the opponent court*

*8: The ball bounces out and behind the service line*

*9: The ball bounces out of right singles sideline, inside the right tram (seen from the position of the return player) or farther away from it*

*10: The ball bounces out of left singles sideline, inside the left tram (seen from the position of the return player) or farther away from it*


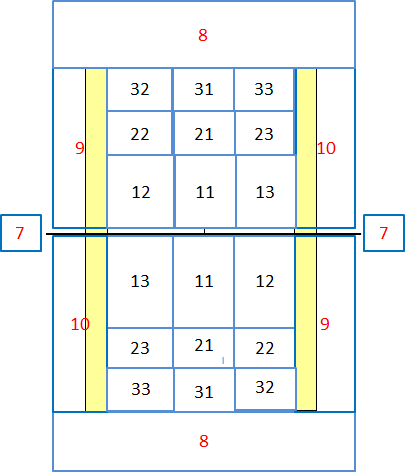


Figure 2. Bounce area after player stroke *(the numbers in red color correspond to the áreas consider like errors)*

- 1. Variables related with the hitting area (Hit_Ar) (FIGURE 3): Corresponds to the area of the court in which the racket of the player impact the ball during the stroke. All areas are exclusive.

| 11 | Behind from the baseline in the central area |
| --- | --- |
| 12 | Behind from the baseline in the right area |
| 13 | Behind from the baseline in the left area |
| 31 | Inside the court and behind of serve line in the central area |
| 32 | Inside the court and behind of serve line in the right area |
| 33 | Inside the court and behind of serve line in the left area |
| 41 | Between the service line and the net in the central area |
| 42 | Between the service line and the net in the right area |
| 43 | Between the service line and the net in the left area |


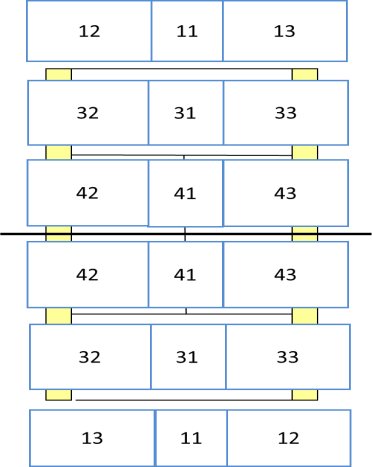


Figure 3. Hitting area

- 1. Variables related with the stroke effectiveness (St_Eff): It make it reference to the outcome of the stroke executed by the player in terms of its effectiveness.

| 1 | Winner |
| --- | --- |
| 2 | Transition stroke |
| 3 | Previous stroke of an opponent error |
| 4 | Error |

*1: Winner: Stroke made by the player with the one that gets the point directly, without his/her opponent touched the ball.*

*2: Transition stroke: Stroke made by player after that, the opponent hit the ball and bounce inside the court of the first one.*

*3: Previous stroke of an opponent error: Stroke made by player after that, the opponent hitting the ball and committed an error losing the point.*

*4: Error: The player hit the ball sending out of the regulatory area of the court or to the net point.*
